# Effect of Recycling Protocol on Mechanical Strength of Used Mini-Implants

**DOI:** 10.1155/2014/424923

**Published:** 2014-07-17

**Authors:** Sérgio Estelita, Guilherme Janson, Kelly Chiqueto, Eduardo Silveira Ferreira

**Affiliations:** ^1^Department of Orthodontics, Faculty of Dentistry, Federal University of Rio Grande do Sul, Rua Ramiro Barcelos, 2492 Bairro Santana, 90035-003 Porto Alegre, RS, Brazil; ^2^Department of Orthodontics, Bauru Dental School, University of São Paulo, Alameda Octávio Pinheiro Brisolla, 9-75 Vila Universitária, 17012-901 Bauru, SP, Brazil; ^3^School of Dentistry, Sao Leopoldo Mandic, Rua Caiubi, 181 Perdizes, 05010-000 São Paulo, SP, Brazil

## Abstract

*Purpose*. This study evaluated the influence of recycling process on the torsional strength of mini-implants. *Materials and Methods*. Two hundred mini-implants were divided into 4 groups with 50 screws equally distributed in five diameters (1.3 to 1.7 mm): control group (CG): unused mini-implants, G1: mini-implants inserted in pig iliac bone and removed, G2: same protocol of group 1 followed by sonication for cleaning and autoclave sterilization, and G3: same insertion protocol of group 1 followed by sonication for cleaning before and after sandblasting (Al_2_O_3_-90 *µ*) and autoclave sterilization. G2 and G3 mini-implants were weighed after recycling process to evaluate weight loss (W). All the screws were broken to determine the fracture torque (FT). The influence of recycling process on FT and W was evaluated by ANOVA, Mann-Whitney, and multiple linear regression analysis. *Results*. FT was not influenced by recycling protocols even when sandblasting was added. Sandblasting caused weight loss due to abrasive mechanical stripping of screw surface. Screw diameter was the only variable that affected FT. *Conclusions*. Torsional strengths of screws that underwent the recycling protocols were not changed. Thus, screw diameter choice can be a more critical step to avoid screw fracture than recycling decision.

## 1. Introduction

Skeletal anchorage with bone screws has been more and more incorporated into orthodontic practice making treatment quicker due to reduced need for patient compliance with anchorage reinforcement appliances. However, bone screw success rate is around 85%, making screw reinsertion an unexceptional clinical event [1–4]. Reinsertion rate can still be greater if it is considered that some orthodontic mechanics require screw repositioning according to the phase of malocclusion correction [5, 6]. Because bone screw anchorage frequently includes screw replacement and repositioning, its reuse has been considered by some authors [5, 7–9], but this clinical procedure can require a careful screw recycling process, which should keep the mechanical and biological characteristics of the bone screws.

An efficient cleaning and sterilization process has to precede bone screw reuse to avoid contamination and infection. The purpose of cleaning is to remove or reduce visible smears including blood, protein, and debris that were precipitated on the screw surface [10, 11]. Sterilization serves to eliminate or stop reproduction of microorganisms including bacteria, spores, and fungi. Usually, cleaning is done first, followed by sterilization. Cleaning processes including electrolytic, ultrasonic, and chemical methods have been used separately or in association to achieve a greater efficiency [11–13]. It has been demonstrated that autoclave sterilization does not influence mechanical resistance, fracture risk, and primary stability of bone screws [13–16]. However, more recently, microscopic images showed that the bone and organic tissues adhered to the surface of failed bone screws were not easily removed even after many cleanings and sterilizations [7]. This highlights that a stricter guideline seems to be needed regarding recycling of retrieved bone screws, mainly if reuse is to be performed between different patients [7]. Furthermore, bone screw surface irregularities, such as scratches produced by the mechanical machining process, roughness associated with sandblasting, and corrosion areas, can make the cleaning process still harder [17–19].

It can be speculated that a more aggressive mechanical cleaning process including a slight abrasive stripping of the screw surface by sandblasting could have a greater efficiency to remove any organic or inorganic residual, providing a safer reconditioning method for used bone screws. However, the impact of this recycling protocol on bone screw performance must be evaluated. Thus, the objective of this study was to evaluate the null hypothesis that the mechanical strength of bone screws retrieved from iliac bone pig and recycled by sandblasting and ultrasonic bath is not different from new screws.

## 2. Materials and Methods

Two hundred bone screws of the same type and brand especially manufactured for this experiment (Dentos, Daegu, South Korea) such that the nominal and core diameter changes were the only variable among them were used in this study. Thus, the dimensional characteristics of head and thread (length, pitch, flank angle, thread form, thread depth, and taper) were systematically standardized because they could distort the effect of the recycling process on the screw mechanical strength, reducing reliability of the results. The sample was divided into four groups with 50 bone screws each, equally distributed in 5 diameters that progressively increased from 1.3 to 1.7 mm, in increments of 0.1 mm. Bone screws of each diameter were randomly allocated in the experimental and control groups.

**Figure 1:**
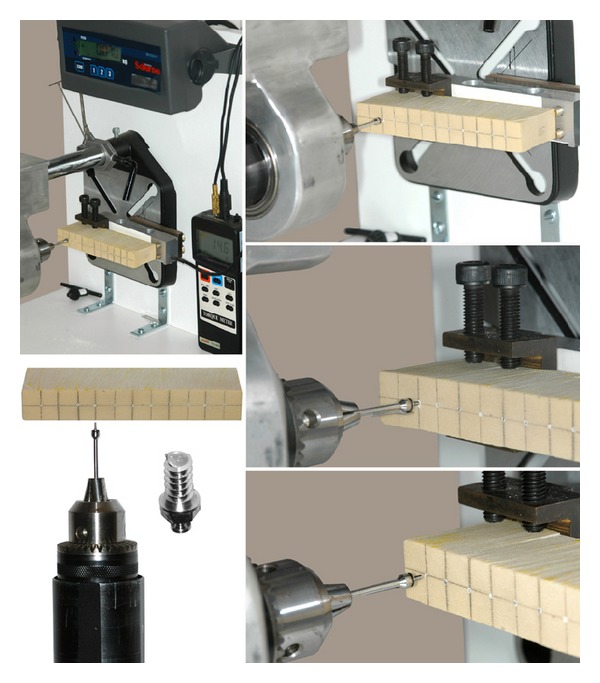
The equipment used for bone screw insertion in high-density artificial bone (0.80 g/cm^3^) and fracture.

Fifty new bone screws (control group, CG) were inserted in high-density artificial bone (0.80 g/cm^3^, Sawbones Division of Pacific Research Laboratories, Vashon Island, Wash, ASTM F1839-08) to achieve a torque value able to cause torsional fracture of the screws (Fracture Torque, FT). An essay machine equipped with a screwdriver fit to a digital torquimeter was used for bone screw insertion, perpendicularly to artificial bone blocks, to achieve fracture torque values that were measured in Newtons per centimeter (Figure 1).

**Figure 2:**
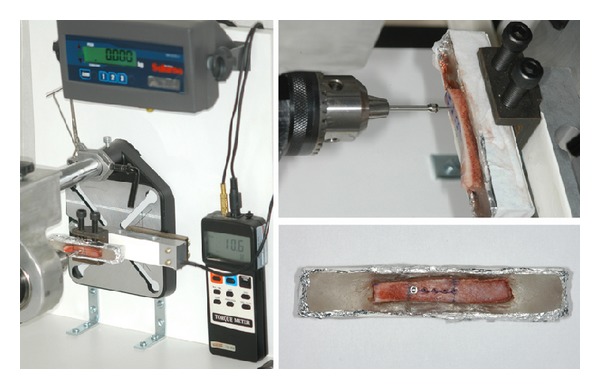
Bone screws inserted into blocks of pig iliac bone. After removal, the bone screws were fractured in high-density artificial bone using the same protocol showed in Figure 1.

Group 1 consisted of bone screws inserted into blocks of pig iliac bone and subsequently removed to reproduce torsional stress developed at the screw threads during clinical placement and removal from the jaw bones (Figure 2). The cortical thickness close to the iliosacral joint ranged from 0.5 to 1 mm, which is similar to the buccal cortical thickness in some anatomic regions of the human maxilla and mandible [20]. After removal, the bone screws were fractured in high-density artificial bone using the same protocol applied to the CG, and fracture torque was recorded.

Bone screws from group 2 were submitted to the same experimental protocol applied to group 1, but before screw insertion into high-density artificial bone for fracture torque measurement, the anchorage devices underwent a recycling process that included ultrasonic cleaning and autoclave sterilization. Ultrasonic bath was operated at 40 kHz and 25°C for 20 min in detergent solution. Afterwards, the screws were rinsed in deionized water and sonicated again for 15 min in deionized water. Subsequently, the bone screws were packed in sealed bags and the autoclaving process was performed at 121°C and 18 psi for 20 min.

**Figure 3:**
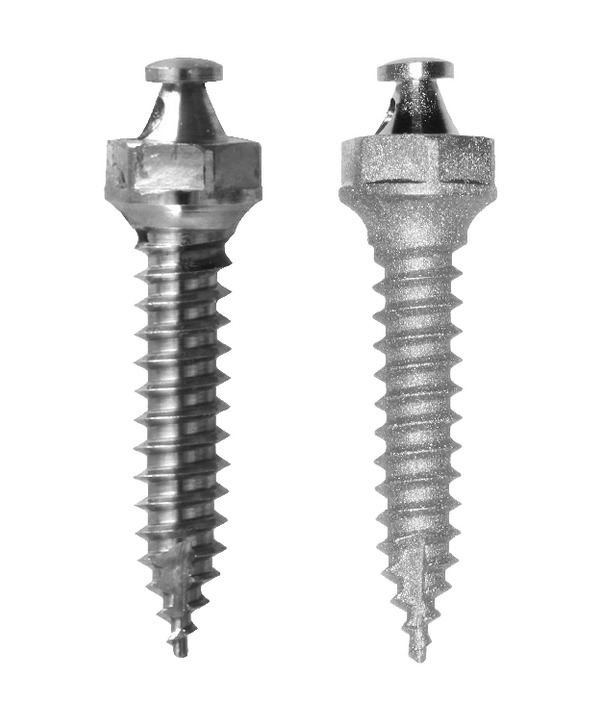
Nonsandblasted and sandblasted bone screws.

Group 3 underwent the same experimental procedures applied to group 2, except for the recycling process of the bone screws, which included sandblasting (Figure 3). The sequence of procedures included ultrasonic bath in detergent solution, rinsing in deionized water, blasting of the screw thread surface with Al_2_O_3_-90 *μ*m particles at 60 psi with the sandblaster unit positioned 10 mm away from the screw surface, and ultrasonic cleaning of the residual alumina particles in deionized water for 20 min.

After the recycling process and before the fracture procedure, the bone screws of groups 2 and 3 were individually weighed on a precision scale to evaluate if a significant amount of metallic structure was lost during sandblasting.

## 3. Statistical Analyses

Analysis of variance (ANOVA) followed by Tukey tests was used to compare the fracture torque among groups and diameters. Weights of the bone screws after the recycling processes were compared between groups 2 and 3 with Mann-Whitney tests. A multiple linear regression analysis was performed taking into account the fracture torque as dependent variable to simultaneously evaluate the influence of two recycling processes, 5 different diameters, and weight loss on the mechanical strength of bone screws. Statistical analyses were performed with Statistica Software (Statistica for Windows 6.0, Statsoft, Inc., Tulsa, Oklahoma), at *P* < 0.05.

## 4. Results

Table 1 shows that the bone screws of the control and experimental groups had similar fracture torque regardless of previous insertion in bone tissue and recycling process applied to groups 1, 2, and 3. However, the fracture torque was significantly greater for each 0.1 mm added in bone screw diameter regardless of previous insertion or recycling protocol.

The weights of sandblasted bone screws were significantly smaller than those of the nonsandblasted (Table 2).

When all the variables (recycling protocols, diameters, and weight loss) were simultaneously evaluated in the regression analysis, bone screw diameter was the only significant variable in predicting fracture torque, explaining more than 97% of its variability (Table 3).

## 5. Discussion

Fifteen to twenty percent of bone screws are early discarded only because of stability failure, which can occur a few days or months after insertion [1, 2, 4]. Other significant percentages of bone screws are early discarded only because the orthodontic mechanics require screw repositioning in the arch to be continued [5, 6]. In both situations, bone screws are frequently thrown away before complete anchorage objectives are met. Thus, it is not surprising that timely bone screw reuse has been considered by orthodontists, reducing the number of titanium screws necessary to finish skeletally anchored treatments [5–9]. However, when bone screw reuse is considered, an initial concern is whether the mechanical strength will support the reinsertion and removal procedures. A similar fracture torque of new (CG) and used bone screws (G1) showed that screws were not weakened after single insertion and removal in pig iliac bone (Table 1). These findings are in accordance with Noorollahian et al.'s [13] study, which found similar fracture torque for unused and single used bone screws. In fact, Defino et al. [21] observed that orthopedic bone screws had the mechanical performance affected only after the third repeated insertion. However, this is only a first condition for screw reuse because, besides structural strength, reuse requires a recycling protocol to produce a reconditioned screw surface free of any organic residuals, microorganisms, or corrosion products.

Several studies have demonstrated that autoclave sterilization does not have any negative impact on bone screw strength and fracture torque even after several sterilization cycles (up to 50 times, as in Adelson et al.'s [15] study) [13–16]. However, some studies have demonstrated that autoclaving alone can fail to completely decontaminate infected instrument if it is not adequately cleaned prior to the sterilization cycle [22, 23]. In general, cleaning can be performed by manual scrubbing, enzymatic agents, and ultrasonic bath, but the association between them (mechanical and chemical regimens) seems to be the most effective cleaning procedure [24, 25]. However, it can be speculated that ultrasonication followed by autoclave sterilization would weaken the bone screw structure; this was not confirmed by the results of this study because a similar fracture torque was observed between control group and group 2 (Table 1).

Importantly, scientific lines of evidence have shown that traditional cleaning processes, such as sonication, even when associated with chemical agents, cannot be sufficient to efficiently remove the proteinaceous biofilm from contaminated instruments, and more aggressive and complex recycling methods as electrolysis have been suggested [7, 12, 26]. Considering that sandblasting is a simple procedure able to clean the screw surface by abrasive mechanical stripping, which can still benefit bone tissue response [27, 28], this study evaluated the mechanical strength of screws previously inserted in pig iliac bone that underwent recycling processes including sonication, sandblasting, and autoclave sterilization (group 3). The results showed that the recycled bone screws had similar fracture torque when compared to the control group and groups 1 and 2 (Table 1). The weights of sandblasted screws were slightly smaller than nonsandblasted, highlighting that sandblasting produced cleaning by abrasive stripping of the superficial layer of the titanium screws with some metallic structure reduction (Table 2) [28]. Nevertheless, this structural loss was limited and not enough to significantly affect the bone screws torsional strengths (Tables 1 and 2).

Only screw diameter variation (0.1 mm) was sufficient to significantly change the fracture torque (Table 1). This fact shows that, from the viewpoint of mechanical strength, the professionals should be more concerned with any screw diameter change, even if small, than if the bone screw was recycled to be reused (Table 1). However, most professionals do not show a great concern if bone screw diameter has to be changed in only 0.1 mm, but they are deeply worried about mechanical performance of recycled bone screws. When the recycling protocols of groups 2 and 3, the five different diameters, and the weight loss were included in a regression model, the only variable significantly and strongly associated with fracture torque was the screw diameter (Table 3). Thus, the choice of screw diameter is critical to perform a safe insertion procedure because an increase in cortical bone thickness can easily approximate the insertion torque from the fracture torque. According to these results, the breakage risk of a reused bone screw is more associated with inadequate diameter choice than with a single recycling process. However, if the screws underwent sequential recycling cycles for multiple uses other results can be found [21].

It has been demonstrated that surface roughness of titanium screws produced by macrosandblasting (Al_2_O_3_-350 *μ*m) has a better response of bone tissues than microsandblasted surfaces (Al_2_O_3_-50 *μ*m). Thus, the evaluated recycling process with Al_2_O_3_-90 *μ*m can still be adjusted to benefit the biological response [27]. Ultrasonic bath in group 3 was used after sandblasting to remove loose particles of Al_2_O_3_ because Al ions could elicit an inflammatory response and disturb bone differentiation and deposition, although this speculation has not been scientifically confirmed [28–30]. Thus, any residual particle of Al_2_O_3_ that remains embedded in the screw surface after recycling does not have deleterious effect on bone response. Instead, some studies consider that Al_2_O_3_ exerts a favorable influence on bone formation [30, 31]. As a consequence, osseointegration and stability of the mini-implant can be benefitted without compromising its removal [32].

This paper provides some guidelines for bone screw recycling, but it does not evaluate professional preference or acceptance degree about this procedure. However, if screw reuse is performed by some professionals, and it is, then this study corroborates the important opinion of other authors that recycling should follow stricter guidelines [7, 12]. From a viewpoint of screw mechanical characteristics, recycling by ultrasonic-sandblasting and autoclave sterilization can be a feasible protocol. However, these results should be complemented by* in vivo *studies because metal corrosion, orthodontic or orthopedic loading, and occlusal forces during chewing can produce an additional impact on mechanical properties of retrieved bone screws.

## 6. Conclusion

Bone screws were not weakened after insertion and removal from pig iliac bone. The recycling protocols did not influence torsional strength of bone screws even when ultrasonic bath was associated with Al_2_O_3_ blasting. Sandblasting cleaning produced an abrasive mechanical stripping of the screw surface, but the structural loss was not sufficient to significantly influence the fracture torque. However, differences of 0.1 mm in bone screw diameter significantly changed the fracture torque.

## Figures and Tables

**Table 1:** Comparison of fracture torque among diameters and recycling groups (ANOVA).

Groups	1.3 mm		1.4 mm		1.5 mm		1.6 mm		1.7 mm		*P*
	Mean	SD	Mean	SD	Mean	SD	Mean	SD	Mean	SD	
CG	9.05^a^	0.53	12.17^b^	0.32	16.45^c^	0.41	21.61^d^	0.51	27.26^e^	0.88	<0.001
1	8.94^a^	0.42	12.21^b^	0.48	16.28^c^	0.73	21.41^d^	0.96	26.98^e^	1.41	
2	8.01^a^	0.60	12.52^b^	0.97	15.75^c^	1.14	21.78^d^	0.47	26.96^e^	1.01	
3	8.04^a^	0.41	11.58^b^	0.39	15.79^c^	0.81	21.17^d^	0.47	26.72^e^	0.72	

*Different letters (e.g., CG row—a, b, c, d, and e) represent significant difference of fracture torque among diameters, defined by Tukey tests.

*Same letters (e.g., 1.3 mm column—a, a, a, and a) represent similarity of fracture torque among groups, defined by Tukey tests.

**Table 2:** Weight comparison between nonsandblasted and sandblasted bone screws (Mann-Whitney tests).

Diameters	Group 2 (nonsandblasted)		Group 3 (sandblasted)		*P*
	Mean (g)	SD	Mean (g)	SD	
1.3 mm	0.0519	0.000568	0.0508	0.000422	0.0014
1.4 mm	0.0567	0.000483	0.0551	0.000568	0.0003
1.5 mm	0.0607	0.000675	0.0599	0.000738	0.0211
1.6 mm	0.0664	0.000516	0.0653	0.000675	0.0040
1.7 mm	0.0746	0.000516	0.0733	0.000823	0.0031

**Table 3:** Influence of recycling protocols, diameters, and weight loss on fracture torque (multiple linear regression analysis).

Independent variables	Beta	SE	*R* ^ 2^	*P*
Recycling protocols	0.025	0.014	<0.001	0.081
Diameters	0.989	0.014	0.979	<0.001
Weight loss	0.008	0.015	<0.001	0.562
